# Application of Cognitive-behavioural Techniques on Changes in the Scope of Dieting Self-efficacy Level among Obese People

**Published:** 2017-01

**Authors:** Malgorzata OBARA-GOLEBIOWSKA

**Affiliations:** Dept. of Psychology of Development and Education, University of Warmia and Mazury, Olsztyn, Poland

## Dear Editor-in-Chief

Weight loss requires a change in eating habits. In most cases, people successfully lost weight regain it after some time. For this reason, obesity-related research programs increasingly often focus on psychological factors that make lifestyle changes and weight reduction maintenance difficult. Self-efficacy is one of the key determinants of weight loss maintenance (1). Dieting self-efficacy (DIET-SE) is defined as individual’s belief in ability to adhere to diet in order to lose or maintain actual weight (2).

Self-efficacy’s impact was dedicated on weight reduction process prove that self-efficacious individuals believe that they can overcome challenges and obstacles while dieting. Therefore, they show more effort and stronger persistence to their slimming related goals adhering. Those people also present better coping strategies because of initiation of behavioral change and learning from their experience to manage better difficult situations. Besides, after breaking their diets self-efficacious individuals recover more quickly and better adhere to their previous goals (3).

In view of the growing prevalence of obesity in the world (3), the objective of this study was to investigate the impact of participation in interdisciplinary organized weight loss programs employing cognitive-behavioural techniques aimed at the development of dieting self-efficacy in obese people.

The study was conducted on 75 obese people (BMI>30), 65 women and 10 men aged 24–65 yr, enrolled in a weight loss program at the Obesity Clinic in Municipal Hospital in Olsztyn, Poland. The participation in the weight loss program involved two wk of cooperation of patients with an interdisciplinary team composed of a psychologist, dietician, physician, and physiotherapist. The participated patients in-group and individual therapeutic activities employing cognitive-behavioural strategies aimed at the development of self-efficacy in the scope of observing a diet.

The measurement tool applied in the study was a scenario-based measure, DIET-SE scale (4). Situations in which obese individuals participate in the study particularly lacked self-efficacy. According to the diagnosis made by DIET-SE, it was implemented in a weight loss program applying cognitive-behavioural strategies. Two measurements of DIET-SE were performed among the participants of the study. The first measurement was performed at the beginning of the weight reduction program, and the second one after the completion of the participation in the two-wk weight reduction program. The results of the study revealed that relatively low initial DIET-SE in the participants and significantly higher levels of DIET-SE among obese people after their participation in a two-wk weight reduction program:

- M1 (first measurement–DIET-SE overall scale) =1.83 (0.55); M2 (second measurement-DIET-SE overall scale) =2.26; t (74) = −8.25; *P*<0.05.
- M1 (first measurement HCF subscale of DIETSE) =1.88 (0.87); M2 (second measurement HCF subscale of DIETSE) =2.54 (1.08); t (74) = −4.63; *P*<0.05.
- M1 (first measurement SIF subscale of DIETSE) =1.91 (0.57); M2 (second measurement SIF subscale of DIET-SE) =2.15 (0.89); t (74) = −5.88; *P*<0.05.
- M1 (first measurement NEE subscale of DIETSE) =1.69 (0.97); M2 (second measurement NEE subscale of DIETSE) =2.26 (0.56); t (74) = −8.25; *P*<0.05.

The efficiency and justifying the application of the cognitive-behavioural therapy were confirmed in treatment of obesity (5). Besides, lifestyle interventions involving cognitive and behavioral techniques for obesity could produce significant improvement of eating self-regulatory skills and, therefore, reductions in weight (6).

Participation in an interdisciplinary weight reduction program employing cognitive-behavioural techniques causes a considerable increase in DIET-SE. Conclusion from the present study have an important practical aspect of obesity treatment because DIET-SE is one of the most important psychological factors both at the stage of implementation of changes necessary for weight reduction and during maintenance of the change and prevention of recurrences (7). Therefore, the application of cognitive-behavioural therapy for the purpose of improvement of DIET-SE of obese patients should be considered in complex treatment of obesity.
